# Examining lysyl oxidase-like modulation of collagen architecture in 3D spheroid models of idiopathic pulmonary fibrosis via second-harmonic generation microscopy

**DOI:** 10.1117/1.JBO.26.6.066501

**Published:** 2021-06-18

**Authors:** Darian S. James, Christopher J. Brereton, Donna E. Davies, Mark G. Jones, Paul J. Campagnola

**Affiliations:** a University of Wisconsin–Madison, Department of Biomedical Engineering, Madison, Wisconsin, United States; b University of Southampton, Clinical and Experimental Sciences, Faculty of Medicine, Southampton, United Kingdom; c University Hospital Southampton, National Institute for Health Research Southampton Biomedical Research Centre, Southampton, United Kingdom; d University of Southampton, Institute for Life Sciences, Southampton, United Kingdom

**Keywords:** second-harmonic generation, collagen, crosslinking, stiffness, polarization

## Abstract

**Significance:** Idiopathic pulmonary fibrosis (IPF) patients have a poor prognosis with short lifespan following diagnosis as there are limited effective treatment options. Despite matrix stiffening being the hallmark of the disease there remains a lack of knowledge surrounding the underlying collagen alterations in the disease. Specifically, while increased collagen crosslinking has been implicated, the resulting effects on collagen macro/supramolecular changes have not been explored.

**Aim:** We sought to determine if second-harmonic generation (SHG) microscopy could characterize differences in the collagen architecture in 3D spheroid models of IPF grown under different crosslinking modulation conditions (promotion and inhibition).

**Approach:** We used SHG metrics based on the fiber morphology, relative SHG brightness, and macro/supramolecular structure by SHG polarization analyses to compare the structure of the IPF spheroids.

**Results:** Comparison of the fiber morphology of the spheroids showed that the control group had the longest, straightest, and thickest fibers. The spheroids with crosslink enhancement and inhibition had the highest and lowest SHG conversion efficiencies, respectively, consistent with the resulting harmonophore density. SHG polarization analyses showed that the peptide pitch angle, alignment of collagen molecules, and overall chirality were altered upon crosslink modulation and were also consistent with reduced organization relative to the control group.

**Conclusions:** While no single SHG signature is associated with crosslinking, we show that the suite of metrics used here is effective in delineating alterations across the collagen architecture sizescales. The results largely mirror those of human tissues and demonstrate that the combination of 3D spheroid models and SHG analysis is a powerful approach for hypothesis testing the roles of operative cellular and molecular factors in IPF.

## 1 Introduction

Idiopathic pulmonary fibrosis (IPF) is a fatal lung disease characterized by excessive scarring and stiffening of the lungs, ultimately resulting in respiratory failure. While IPF etiology is poorly understood, it has been attributed to a non-specific combination of both genetic and environmental factors, which promote repetitive alveolar injuries.^1^^–^^3^ Upon injury, alveolar epithelial cells are aberrantly activated and secrete profibrotic cytokines, especially transforming growth factor-beta (TGF-β).^3^ Fibroblast activation by TGF-β results in a highly contractile and myofibroblast phenotype, which serves as the primary effector cell for increased collagen (and other matrix proteins) production and extensive tissue remodeling.^3^^,^^4^ Despite being the hallmark of the disease, the collagen architectural alterations themselves have not been well studied. Specifically, there have been limited TEM imaging studies and cellular level imaging has been confined to H&E pathology, where the limitations of the latter are well-documented.^5^ Further examination of the operative cell-matrix interactions could provide better insight into disease etiology and also prognosis. Moreover, while there are now two FDA-approved drugs to treat IPF (pirfenidone and nintedanib), their mechanisms are not well understood, and better assessments of their efficacy would be helpful in defining treatment regimens. Because of these collective difficulties, there remains a clear need for both better diagnostic techniques as well as an understanding of the extracellular matrix (ECM) changes that occur in the disease.

While the role of several species (e.g., TGF-β and ROCK) have been well-studied at the cellular level, their effects on modulation of the collagen architecture remain largely unknown. We have previously utilized second-harmonic generation (SHG) microscopy to probe the collagen structure in IPF and delineated it from normal tissue by different means.^6^^,^^7^ Specifically, we used machine learning to classify normal and IPF human lung tissues with high accuracy (∼95%) based on the collagen morphology.^6^ More recently, we used polarization-resolved SHG as well as the SHG emission directionality (forward/backward measurements) to examine the macro/supramolecular changes and found that the collagen was more disorganized at both the triple helical and fibril levels of structure.^7^ Interestingly, similar trends were also seen in human ovarian cancer, which is characterized by up-regulation of several of the same pathways as IPF.^8^^–^^10^

While we have documented these changes in IPF, it is important to elucidate the underlying molecular factors that give rise to these alterations. Here, we begin this process by examining the role of crosslinking modulation on the collagen architecture. Lysyl oxidase (LOX) and its counterparts, lysyl oxidase-like (LOXL) proteins, are responsible for crosslinking collagen and elastin in the ECM and are required for the structural integrity of many tissues.^11^^–^^14^ Studying LOX and LOXL induced changes on collagen structure is potentially relevant for IPF, as increased crosslinking results in tissue stiffening, which is a hallmark of the disease.^4^ Indeed, Jones and Tschumperlin have shown that LOXL family members are up-regulated in *ex vivo* IPF tissues.^4^^,^^15^

Examining the impact of crosslinking on the collagen architecture using well controlled *in vitro* models can yield new insight into the disease progression as this process can be modulated through selective promotion and inhibition. For example, using long term spheroids (40 to 60 days), Jones recently showed that increased bone type pyridinoline collagen crosslinking, mediated by lysyl hydroxylase 2 (LH2/PLOD2), LOXL2, and LOXL3 altered nano-scale architecture.^4^ Importantly, selective inhibition normalized mechano-homeostasis and limited the self-sustaining effects on fibrosis progression.

While this study focused on the biomechanics aspects, initial SHG imaging also revealed alterations in the collagen fiber morphology. Here, we extend the characterization of the collagen architecture in similar IPF spheroid models with selective crosslinking modulation by further analysis of fiber morphology, examination of coherence aspects through SHG conversion efficiency, as well as probing macro/supramolecular changes through polarization-resolved SHG (P-SHG and SHG-CD) imaging.

## 2 Materials and Methods

### 2.1 *In Vitro* Spheroid Preparation

IPF lung fibroblasts were cultured using the 3D *in vitro* model of fibrosis as previously described.^4^ All human lung experiments were approved by the Southampton and South West Hampshire and the Mid and South Buckinghamshire Local Research Ethics Committees (ref 07/H0607/73), and all subjects gave written informed consent. Briefly, peripheral lung fibroblasts were obtained as outgrowths from surgical lung biopsy tissue of patients (n=3 donors) who were subsequently confirmed with a diagnosis of IPF according to international consensus guidelines.^16^ All primary cultures were tested and free of mycoplasma contamination.

The fibroblasts were seeded in Transwell inserts in Dulbecco’s Modified Eagle Medium (DMEM) containing 10% FBS. After 24 h, the media were replaced with DMEM/F12 containing 5% FBS, 10-μg/ml L-ascorbic acid-2-phosphate, 10-ng/ml epidermal growth factor, and 0.5-μg/ml hydrocortisone with or without N-[[1,2-dihydro-4-hydroxy-2-oxo-1-(phenylmethyl)-3-quinolinyl]carbonyl]-glycine (IOX2) (50  μM, Stratech Scientific) and/or PXS-5120 (10  μM, Pharmaxis, Australia), as indicated. Each experiment included a vehicle control. TGF-$\beta_{1}$ (3  ng/ml) was added to the cultures, and the medium replenished three times per week. After 42 or 60 days in culture, the spheroids were harvested and fixed in 4% paraformaldehyde for imaging analyses or flash frozen for crosslinking analyses. These were then sent to the Campagnola lab for SHG imaging. On average, the spheroids had a full thickness of 400 to 600  μm. However, due to attenuation, we cannot image through the full volume and used a vibratome to slice the spheroids into individual ∼100 μm thick sections, and then, we imaged multiple such sections.

Samples for imaging analyses were stored at 4°C in phosphate-buffered saline (PBS) for conventional SHG imaging or optically cleared by immersion in 50% glycerol overnight to reduce scattering-induced depolarization effects for SHG polarization-resolved imaging. For imaging, samples were mounted on glass slides in PBS or glycerol with #1.5 coverslips, and nail polish was used to seal the slides. A total of 12 independent samples were imaged.

Collagen crosslinks were calculated as previously reported.^4^ Briefly, total mature pyridinium collagen crosslinks [PYD + deoxypyridinoline (DPD)] were determined using an enzyme-linked immunosorbent assay (ELISA; Quidel Corporation, San Diego) according to manufacturer’s instructions. For normalization, total collagen content was estimated by colorimetric assay of hydroxyproline (Hyp) based on the reaction of oxidized hydroxyproline with 4-(dimethylamino) benzaldehyde, as per manufacturer’s instruction (Sigma-Aldrich, Poole). The molar content of collagen was estimated from hydroxyproline using a conversion factor of 300 hydroxyprolines per triple helix, and mass of collagen was estimated using a molecular weight of 300 kDa per triple helix. Quantitation of the collagen crosslinks was achieved by comparison to a standard curve. Sample values were interpolated using GraphPad Prism software.

### 2.2 SHG Microscope System

The details of the SHG microscope have been described elsewhere^17^^,^^18^ and are only briefly described here. The system consists of a laser scanning unit (FluoView 300; Olympus, Melville, New York) mounted on an upright microscope (BX61; Olympus, Tokyo, Japan), where the excitation source is a modelocked Titanium Sapphire laser (Mira; Coherent, Santa Clara, California). SHG imaging was performed with a fundamental laser wavelength of 890 nm for morphology, conversion efficiency, and linear polarization (P-SHG) analysis and 780 nm for SHG circular dichroism (SHG-CD), where the shorter wavelength for the latter provides greater sensitivity.^10^ Average powers at the focus were ∼30 to 50 mW using a 40×0.8 NA water immersion lens (LUMPlanFL; Olympus, Tokyo, Japan) and a 0.9 NA condenser was used for collection of forward SHG. The resulting lateral and axial resolutions were ∼0.7 and 2.5  μm, respectively.

Forward SHG emission was collected using a photon-counting detector (7421 GaAsP; Hamamatsu, Hamamatsu City, Japan). The SHG wavelength (445 and 390 nm) was isolated with the respective 10 nm wide bandpass filters (Semrock, Rochester, New York) and the excitation wavelength was confirmed using a fiber-optic spectrometer (Ocean Optics, Dunedin, Florida). Fields of view were 170×170  μm for non-polarization-resolved SHG and 85×85  μm for both SHG-CD and P-SHG where these were acquired with scanning speeds of 2.71  s/frame with three-frame Kalman averaging. The power was controlled by an electro-optic modulator (ConOptics, Danbury, Connecticut) run by a custom LabVIEW program (National Instruments, Austin, Texas), interfaced with the FluoView scanning system using a data acquisition card (PCI-6024E; National Instruments).

Linear polarization was obtained using a half-wave plate to define the state entering the microscope and the desired linear rotation at the focal plane was achieved using a liquid crystal rotator (LCR; Meadowlark Optics, Frederick, Colorado) mounted in the infinity space.^18^ Circular polarization is achieved with a quarter-wave plate after the LCR, where left- and right-handed states are achieved with 90 deg of linear rotation by the LCR.^18^ The linear and circular polarization states were validated as previously described by imaging cylindrically symmetric giant vesicles.^10^^,^^18^ The polarization control was also run by a custom LabVIEW program interfaced to the FluoView scanning system.

### 2.3 Relative SHG Conversion Efficiency

We determined the relative SHG conversion efficiency, which arises from the collagen concentration and organization, by measuring the forward attenuation, i.e., the rate of SHG intensity decrease with increasing depth into the tissue. We have shown that this response is a coupled effect of the relative conversion and primary filter effects (i.e., loss of laser power) and in general Monte Carlo techniques are necessary to isolate the former.^19^^,^^20^ The spheroids used in this study are less than one scattering length thick and this approach is not necessary for relative intensity measurements. Due to intrinsic heterogeneity in concentration, it is necessary to normalize the SHG intensity response to account for local variability within the same tissue (different fields of view) and to make relative comparisons between different tissues. We normalized each optical section within each optical series and these were self-normalized with the average maximum intensity value. Then, the difference in slope of the attenuation is directly related to the conversion efficiency.^21^^,^^22^ Images were collected for the entire thickness of the spheroids in four different locations.

### 2.4 SHG Polarization Analysis

While the spheroids are not highly scattering, we have shown even one scattering length gives rise to significant depolarization in collagenous tissues.^23^ The scattering length in these models is ∼100  μm where we have found this in other *in vitro* systems (unpublished). Here, spheroids were sliced into sections of about 100  μm in thickness and the optically cleared with 50% glycerol to essentially eliminate these effects. Additionally, images were obtained 20-μm deep into the samples to avoid boundary effects.

#### 2.4.1 Helical pitch angle and anisotropy analysis

Polarization-dependent measurements were performed as previously described,^9^ where images were taken every 10 deg through 180 deg of rotation of the incident laser polarization and SHG signal polarization. The α-helical pitch angle is determined by rotating the laser polarization and measuring the SHG intensity. Then the data were analyzed using the combination of the pixel-based generic model^24^ and the single-axis molecular model.^25^ In accordance with previous work, we determined the α-helical pitch angle, $\theta_{p}$, through analysis of the symmetry reduced tensor elements:

$$\theta_{p}=\tan^{-1}\sqrt{2/b}=\tan^{-1}\sqrt{2/(\chi_{ZZZ}^{\left(2\right)}/\chi_{ZXX}^{\left(2\right)}).}$$ (1)

The SHG signal polarization was further determined on a pixel basis and as a function of laser polarization. The anisotropy, β, is reflective of the alignment of dipole moments within the focal volume. The limiting cases are 0 and 1, representing totally random and perfectly aligned structures, respectively, and is calculated as

$$\beta (\theta )=\frac{I_{Par}^{2\omega }(\theta )-I_{Perp}^{2\omega }(\theta )}{I_{Par}^{2\omega }(\theta )+2I_{Perp}^{2\omega }(\theta )},$$ (2)

where $I_{Par}^{2\omega }$ and $I_{Perp}^{2\omega }$ represent the parallel and perpendicular SHG polarization response, respectively.

#### 2.4.2 Second-harmonic generation-circular dichroism

SHG-CD analysis was used to interrogate the overall chirality of the collagen where the method has been described previously.^10^ To account for variations in intensity in the different spheroids, we report the normalized SHG-CD response defined as

$$I_{SHG-CD}=\frac{\left|I_{(2\omega )LHCP}-I_{(2\omega )RHCP}\right|}{[I_{(2\omega )LHCP}+I_{(2\omega )RHCP}]/2},$$ (3)

where $I_{2(\omega )LHCP}$ and $I_{2(\omega )RHCP}$ represent the integrated pixel intensities of SHG images excited with left-handed circular polarized (LHCP) and right-handed circularly polarized (RHCP) light, respectively. This is calculated on a pixel basis, where we first set a threshold mask above the noise background. Absolute values were summed across the entire field of view as the sign of the CD response will depend on fiber orientation.^10^

### 2.5 Statistical Analysis

One-way analysis of variance with post hoc two-way Student’s t-tests were performed, where p-values less than α=0.05 were considered statistically significant. The statistics toolbox in Origin 9.1 (OriginLab, Northampton, Massachusetts) was used.

## 3 Results

### 3.1 Collagen Fiber Assembly

**Fig. 1:**
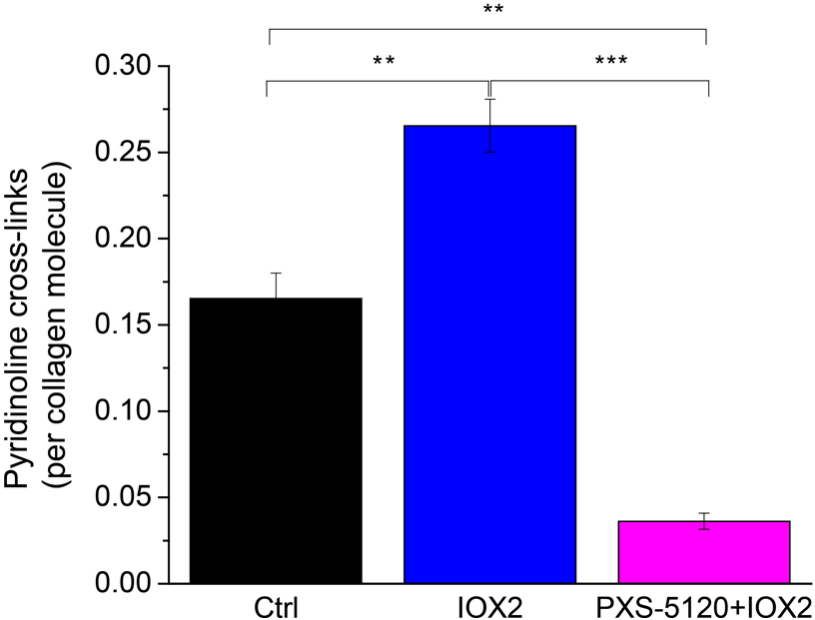
Enhancement or inhibition of pyridinoline crosslinking in a 3D *in vitro* model of fibrosis. Total mature trivalent (PYD + DPD) collagen crosslinks determined by ELISA. n=6 samples from three IPF donors. ** indicates p<0.01 and *** indicates p<0.0001.

We compared the collagen fiber morphology of 42- and 60-day spheroids for the four treatment groups: (i) Control (Ctrl); (ii) PXS-5120 (10  μM, a dose that inhibits all LOX/LOXL enzymes and inhibits pyridinoline collagen crosslinking^4^); (iii) IOX2 (which enhances pyridinoline collagen crosslinking^26^^,^^27^); and (iv) IOX2 + PXS-5120. After 42 days in culture, mature pyridinoline collagen crosslinks (DPD/PYD) were significantly increased in the presence of IOX2, whereas PXS-5120 significantly reduced pyridinoline crosslinks (Fig. 1).

**Fig. 2:**
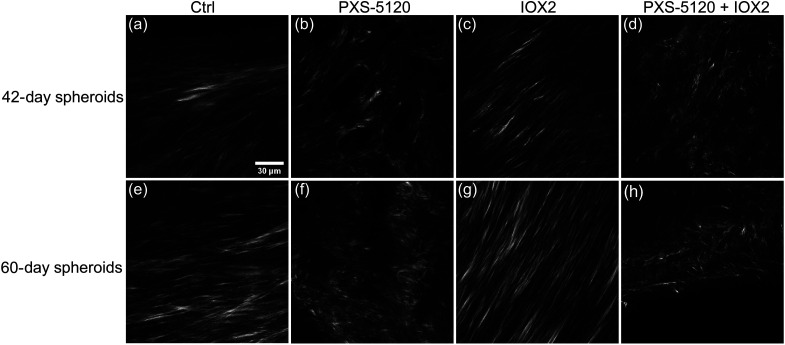
Representative SHG images of *in vitro* IPF samples for 42- (top row) and 60-day (bottom row) cultures. The collagen morphology for the control [(a) and (e)], crosslinking inhibitor [(b) and (f)], crosslinking promoter [(c) and (g)], and inhibitor + promoter [(d) and (h)] treatment groups are shown. Scalebar=30  μm.

Representative SHG images for the four groups are shown in Fig. 2. The control [Figs. 2(a) and 2(e)] and IOX2 treated [Figs. 2(c) and 2(g)] spheroids appear with denser collagen accumulation and brighter SHG intensity in comparison to samples where collagen crosslinking has been inhibited. Notably, spheroids treated with PXS-5120 (including PXS-5120 + IOX2) had shorter and less pronounced fiber structures, whereas the Ctrl and IOX2 collagen morphologies were elongated and straighter for both 42- and 60-day cultures.

**Fig. 3:**
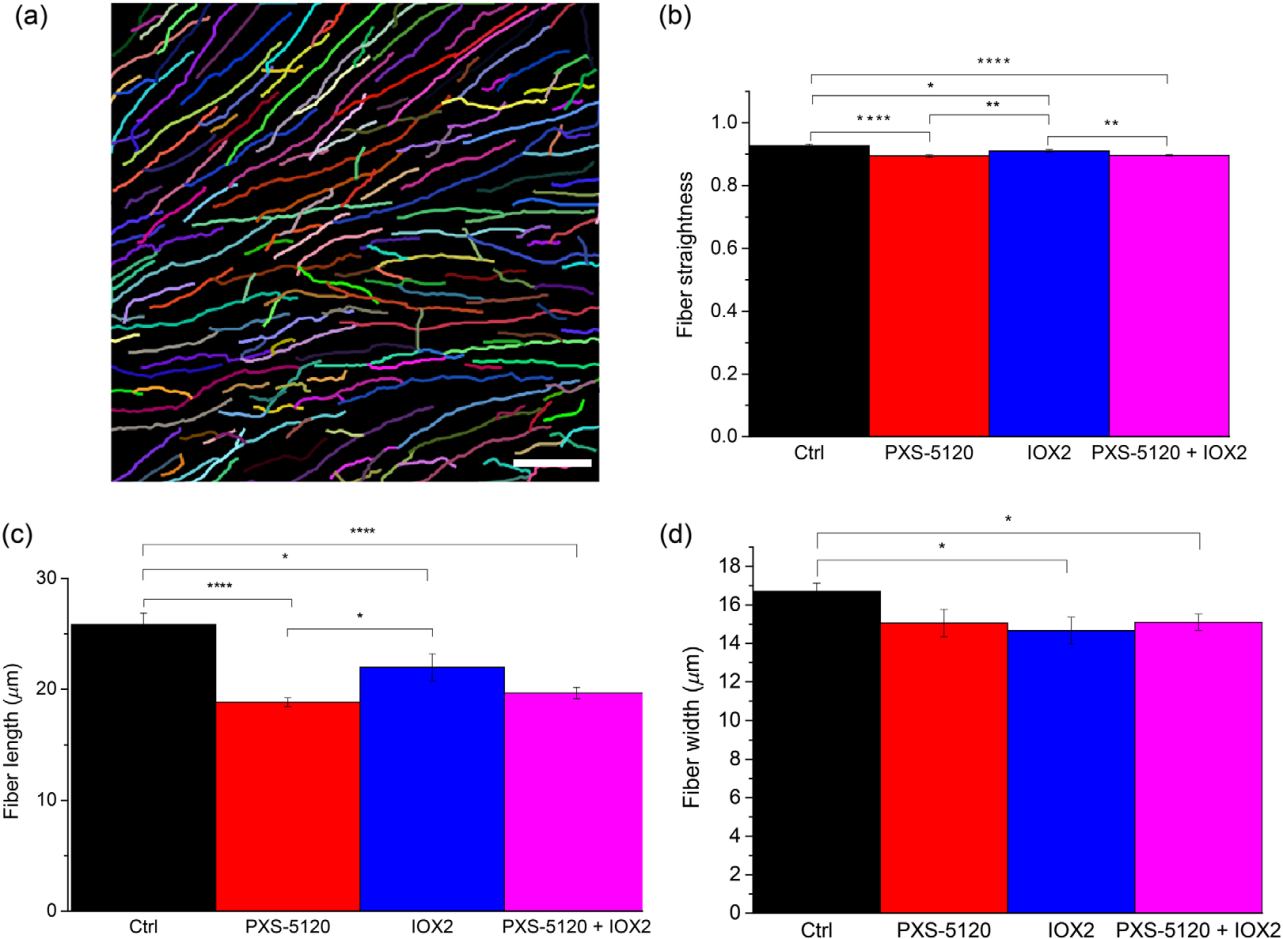
Representative collagen fiber/fiber bundle map. Average collagen fiber straightness, length, and width for control (black), LOXL inhibitor (red), crosslinking promoter (blue), and inhibitor in combination with promoter (magenta) *in vitro* samples quantified by CT-FIRE software. Standard error bars are shown. * indicates p<0.05, ** indicates p<0.01 and **** indicates p<0.00001. Scale bar=30  μm.

We employed curvelet transform-fiber extraction (CT-FIRE)^28^ to quantify these fiber characteristics, where this analysis utilizes both the fast discrete CT and a FIRE algorithm to yield descriptive collagen fiber statistics.^29^ As FIRE was designed to extract fibers from relatively sparse collagen gels, this analysis is sometimes unable to distinguish between individual fibers and fiber bundles. A representative CT-FIRE fiber map of Ctrl is shown in Fig. 3(a).

First, we examined collagen fiber/fiber bundle straightness in the four groups. The Ctrl and IOX2 (enhanced crosslinking) spheroids had the straightest fibers [Fig. 3(b)]. Additionally, the Ctrl group had the longest average fiber length followed by the IOX2 treatment group [Fig. 3(c)], where in contrast the groups with inhibition treatment had shorter fibers. There were also differences in the fiber widths, where the Ctrl group [Fig. 3(d)] was characterized by the thickest fibers. Reduced fiber diameter and length occur upon enhanced crosslinking as the covalent bonds between individual tropocollagen molecules pulls the fibers together. Essentially, the same number of molecules are packed into a smaller space. This is borne out by recent TEM studies.^30^ On the other hand, crosslink inhibition would be expected to decrease length and width due to decreased organization.

**Fig. 4:**
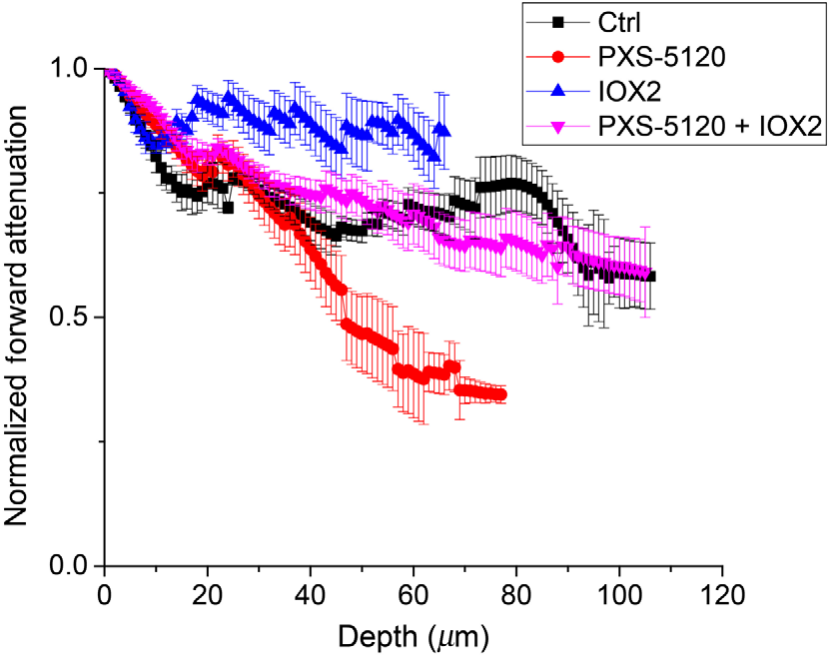
Forward attenuation as a function of depth for control (black), LOXL inhibitor (red), crosslinking promoter (blue), and inhibitor in combination with promoter (magenta) *in vitro* samples. Standard error bars are shown.

To further characterize the effects of collagen crosslink modulation on the fiber architecture, we determined the relative normalized SHG conversion efficiency of each group by measuring the forward attenuation (see Sec. 2.3). The averaged data is shown in Fig. 4, where the normalized rate of decay corresponds to the conversion efficiency, i.e., a flatter depth-dependent response is associated with greater brightness. We found that the IOX2 spheroids had the highest conversion efficiency, whereas the PXS-5120 inhibition had the most rapid decrease in intensity and corresponding conversion efficiency, where the difference between these groups was ∼3 fold, based on the relative values at 80  μm into the spheroid. The Ctrl and promoter/inhibitor groups were similar to each other and were in between the limiting cases.

These results are consistent with our expectations as increased crosslinking should increase SHG brightness due to collagen molecules being more densely packed in fibers. This is because in the limiting case of complete alignment, the SHG efficiency scales as the square of the collagen concentration. Conversely, crosslinking inhibition should result in weaker SHG due to decreased harmonophore density. We note that we performed this analysis on ∼100-μm thick sections as opposed to thin sections (∼5 to 10  μm) as the latter can display significant edge/cutting angle effects and consistent SHG intensities are difficult to obtain.^31^

### 3.2 Polarization-Resolved SHG Analysis

#### 3.2.1 Peptide pitch determination

**Fig. 5:**
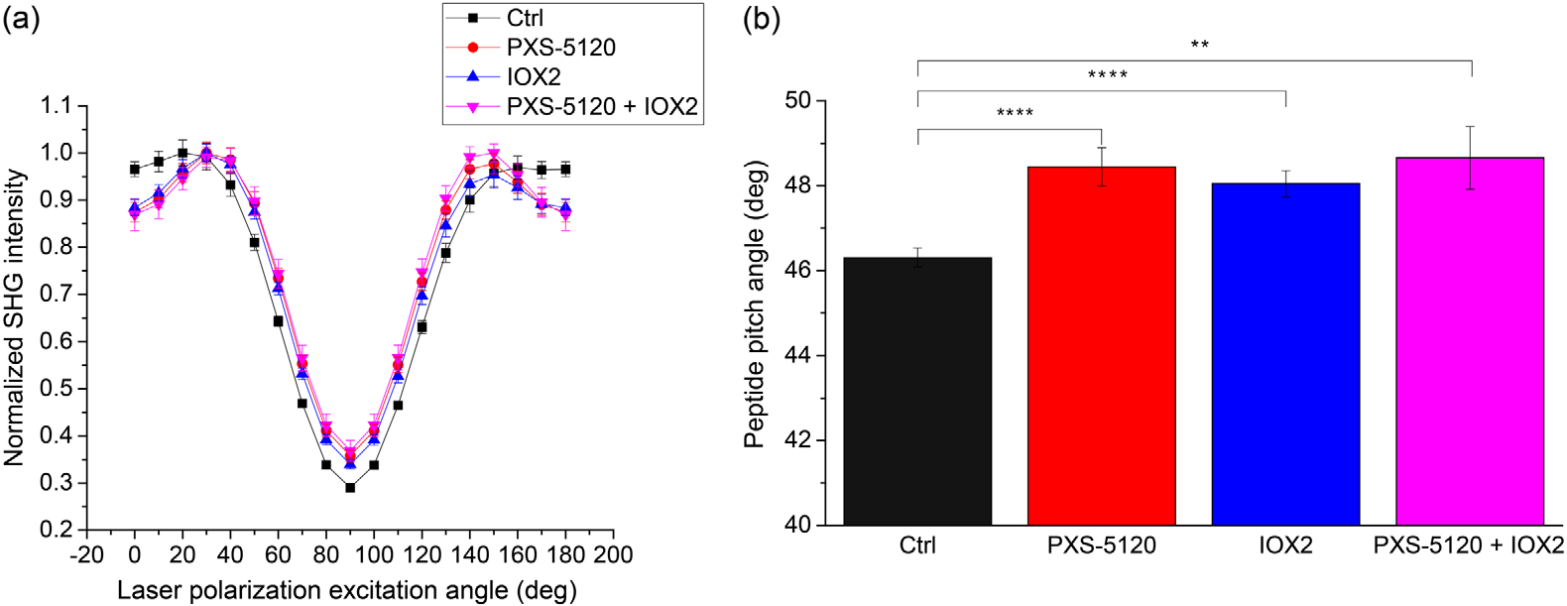
Linear polarization analysis of Ctrl (black), LOXL inhibitor (red), crosslinking promoter (blue) and inhibitor in combination with promoter (magenta) *in vitro* samples, where the reconstructed pixel-based response and the extracted pitch angles are in (a) and (b), respectively. Standard error bars are shown. ** indicates p<0.01 and **** indicates p<0.00001.

By reconstructing the pixel-based SHG intensity as a function of excitation polarization angle using the generic model [Fig. 5(a)] and then fitting to the single-axis molecular model,^25^ we extracted the effective peptide pitch angle for each group [Fig. 5(b)]. The measured pitch angle for the Ctrl spheroid is similar to what we found in normal human lung and other tissues comprised of Col I.^7^^–^^9^ In the spheroids with crosslink modulation, we found larger pitch angles (all significantly different from the Ctrl but not from each other). We do not know the specific molecular changes that lead to the apparent higher angle, but LOX crosslinking binds triple helical and fibril units together and can likely change the underlying effective peptide structure probed by SHG.

#### 3.2.2 SHG signal anisotropy

**Fig. 6:**
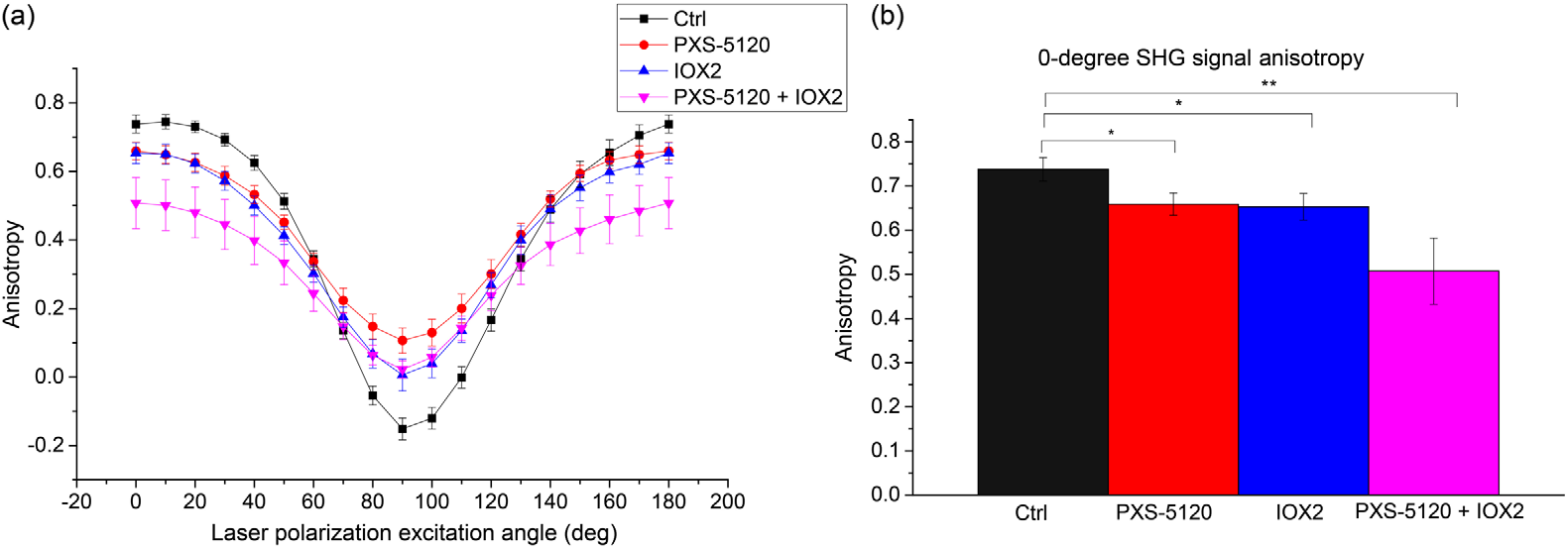
Pixel-based SHG signal anisotropy responses for Ctrl (black), LOXL inhibitor (red), crosslinking promoter (blue), and inhibitor in combination with promoter (magenta) *in vitro* samples. Reconstructed anisotropies at (a) all excitation angles and (b) individual 0-deg angle. Standard error bars are shown. * indicates p<0.01 and ** indicates p<0.001.

We also examined the SHG signal anisotropy, [β; Eq. (2)], which is a measure of the alignment of dipole moments within the focal volume, in the four treatment groups [Fig. 6(a)]. As previously described, this is performed at all angles of excitation and is acquired at the same time as the data for the peptide pitch angle determination (Fig. 5).^9^ The mean anisotropy value for Ctrl spheroids was higher at 0 deg and lower at 90 deg than other treatment groups. Indeed, these values are similar to those we previously reported for collagen in well-aligned tissues^17^ and in Col I gels.^9^ The negative value at 90 deg is non-physical and arises from subtraction errors from pixels with very low signal (near zero) at this orthogonal excitation polarization angle. The groups with crosslinking modulation had lower and higher anisotropies at 0-deg and 90-deg excitation, respectively. The data suggest that LOXL promotion and/or inhibition alters the alignment of dipole moments where they become less aligned within fibrils. For a simple comparison, the extracted values for 0-deg excitation are shown in Fig. 6(b). We note that the sensitivity of this measurement is ∼0.02 and values in the range of ∼0.5 to 0.6 correspond to highly disordered structures relative to normal Col I.

#### 3.2.3 SHG-CD analysis of chirality

**Fig. 7:**
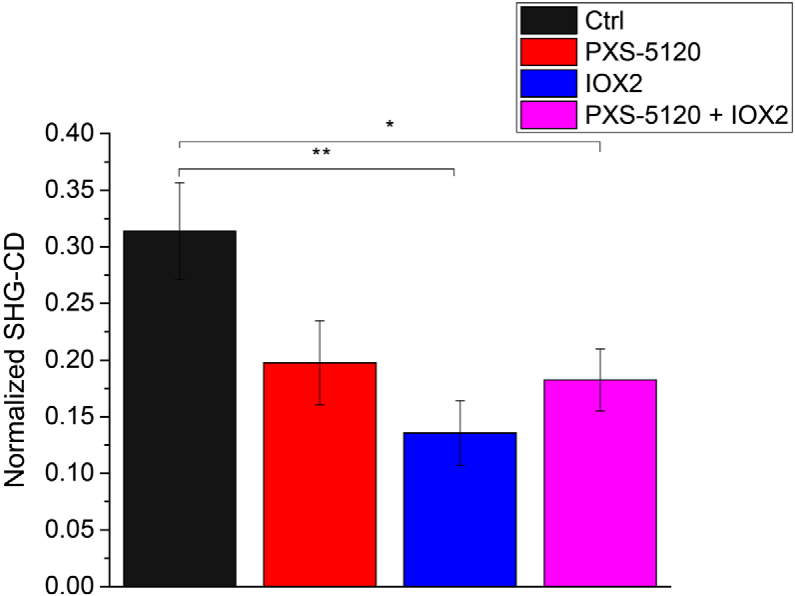
Normalized SHG-CD data of optically cleared Ctrl (black), LOXL inhibitor (red), crosslinking promoter (blue), and inhibitor in combination with promoter (magenta) *in vitro* samples. * indicates p<0.01 and ** indicates p<0.001.

We probed the net chirality of the collagen using the SHG-CD approach described previously.^10^ The normalized SHG-CD responses are shown in Fig. 7 for all treatment groups. We first note that the SHG-CD response is highest for the Ctrl group, suggesting that collagen molecules are aligned primarily on axis in the fibrils. This is similar to what we have seen in well aligned tissues and in collagen gels.^9^^,^^17^ This decrease in collagen chirality in IOX2 spheroids is reflective of decreased alignment of the molecules within the fibrils due to increased collagen crosslinking. Similarly, crosslinking inhibition by PXS-5120 also produces collagen with decreased chirality. While the SHG-CD results are analogous to the anisotropy (Fig. 6), this mechanism does not probe the same structural aspects. Specifically, SHG-CD probes the triple helical structure and how these units are assembled into fibrils, rather than the alignment of the dipole moments.

## 4 Discussion

While previous efforts have probed structural changes in IPF and normal lung architecture via immunofluorescence, AFM, and other techniques,^4^^,^^6^^,^^13^^,^^16^^,^^32^ the impact of collagen crosslinking on the collagen structure has not been well explored. For example, although it has been documented that LOX expression is increased in human IPF,^3^^,^^4^ the resulting macro/supramolecular changes are unknown. Purely structural analyses cannot provide this essential information and spectroscopic approaches are required. For instance, previous studies have examined collagen crosslinking via Fourier transform infrared (FTIR) spectroscopy,^33^ as the amide I band is sensitive to the collagen secondary structure.^34^ However, this technique requires isolation, digestion, or other extensive sample preparation, and FTIR has insufficient resolution. Additionally, the connection between the amide I and II bands and crosslink density has had limited success.

SHG has great potential for this task as we have shown that sub-resolution structural information can be extracted.^7^^–^^9^^,^^35^ However, while the SHG response should be influenced by changes in crosslinking, there are no SHG signatures that are uniquely attributed to these alterations. Others have addressed this problem by imaging pyridinoline crosslinks in gels and tissues via the combination of SHG and two-photon excited fluorescence (TPEF).^36^^–^^41^ However, these measurements can be challenging to assess as the autofluorescence bands can come from different conformations and the spectra can be varied. Quantification is further complicated due to the quadratic dependence of SHG on concentration, whereas TPEF is proportional to crosslink concentration.

As a new approach to examining the effects of crosslinking modulation on collagen architecture, we implemented SHG microscopy using several metrics based on analysis of fiber morphology, conversion efficiency (based on coherence), and polarization responses using *in vitro* models with selective promotion and inhibition. This work builds on the investigations of Jones and coworkers, where using spheroid models and mechanical and biochemical measures, they identified the importance of dysregulated crosslinking in IPF in terms of collagen structure-function.^4^^,^^30^ It is important to study these alterations in models where crosslinking is specifically modulated to fully understand the architecture changes. Notably, these experiments cannot be done on *ex vivo* human tissues, and further, no representative animal models of IPF exist. Thus, the combined use of detailed SHG imaging analyses with well-defined crosslink modulation allows systematic hypothesis testing of biochemical factors that affect structure. Identifying these features in a known *in vitro* system is a critical step for eventual translation to *in vivo* imaging diagnostics. Below we describe the effects on different levels of collagen architecture.

The relative conversion efficiency arises in part from the phase-mismatch, Δk, which is defined by $\Delta k=k_{2\omega }-2k_{\omega }$, where $k_{2\omega }$ and $k_{\omega }$ are the wave vectors for the SHG and incident photons, respectively. Here the SHG intensity is modulated by a sinc^2^ function of Δk, where a smaller phase-mismatch results in higher intensity.^19^ In the current case, a higher local density of collagen molecules will arise upon enhanced crosslinking and will lead to increased SHG intensity over the Ctrl condition as the phase-mismatch will be lower (see Fig. 4). Analogously, crosslink inhibition will increase phase-mismatch and produce weaker SHG contrast. However, this situation is more complicated as the net SHG intensity also depends on the organization, which we probed via polarization-resolved SHG.

None of the three polarization responses (peptide pitch angle, signal anisotropy, and chirality) are uniquely associated with crosslinking. However, these metrics for the control spheroids (Ctrl) are similar to those seen in normal Col I, for example in tendon and collagen gels.^9^^,^^17^ Notably, all these measures are different for all forms of collagen crosslink modulation. While we do not yet know the specific macromolecular changes, all three responses are consistent with decreased organization. Of specific relevance is the SHG signal anisotropy (Fig. 6), as lower values of β correspond to a decrease in molecular alignment, leading to weaker SHG. This effect is analogous to what we previously reported in mixed Col I/Col III self-assembled gels, where increasing fractions of the latter decreased the alignment of the dipoles, as inferred by lower and higher anisotropies and 0-deg and 90-deg excitation, respectively.^9^ There was also a marked difference in the net peptide pitch angle upon crosslinking alterations. We note that while we used this analysis previously to probe the structure of Col I/Col III mixed gels,^8^^,^^9^ the models here were grown only from IPF fibroblasts and we do not expect collagen isoform modulation. However, this measurement does have sensitivity to structural changes upon crosslinking alteration.

We also note a possibly unexpected difference between the decreased anisotropy and increased alignment in the IOX2 treated group. The apparent discrepancy arises from different operative size scales in collagen architecture. The SHG signal anisotropy measurement describes the dipole moment alignment angle within collagen fibrils (formed by crosslinked collagen molecules; 50 to 100 nm in diameter). Collagen molecules are almost perfectly aligned on the long axis in a normal collagen fibril,^42^ and we have shown these become altered in human IPF tissues.^7^ As expected, the dipole moments within the measured volume become less aligned upon crosslinking modulation, where this is consistent with the decreased fiber lengths and widths in Fig. 3. Importantly, we previously showed that the fibers in human IPF tissues were more aligned than in normal tissues,^6^ whereas at the same time the fibrils were indeed less ordered. As many factors affect the SHG response, it is important to use a suite of tools to fully characterize the response.

We also note that SHG-CD (Fig. 7) is related to conventional UV absorption CD in terms of probing chirality, but the underlying mechanisms are different. For example, spectroscopic CD uniquely probes absorption bands associated with secondary (∼190 to 220 nm) and tertiary (∼250  nm) structures, whereas SHG-CD arises from coherence and not absorption and reports the overall chirality as opposed to specific structural aspects. Several factors give rise to the SHG-CD response including out-of-plane tilt angle, sub-resolution molecular alignment, and overall chirality. The direct relationship of tilt and SHG-CD response was rigorously derived by Barzda, where they showed an out-of-plane tilt is essential for a non-vanishing response.^43^ We previously discussed the issue of polarity in SHG-CD, where a normal fiber has an overall handedness along its length with all of the molecules aligned in the same direction or having the same C-N/N-C terminus throughout.^10^ In the current case, the models with crosslink modulation have lower SHG-CD responses, where this may arise from a combination of reduced alignment of dipoles moments in the fibrils, (borne out by the lower SHG signal anisotropy in Fig. 6) and reduced chirality from improper single helices (from pitch angle; Fig. 5) assembling into an improper triple helix. Interestingly, Schanne-Klein and co-workers recently suggested that SHG-CD probes homogeneous polarity versus a mix of antiparallel fibrils,^44^ which agrees with our current studies. Overall, our findings are thus mainly consistent with this report. We note that we are not sensitive to title angle in our measurements.

The decreased chirality along with large changes in the effective pitch angle (Fig. 5) are consistent with large structural changes. Additionally, the decreased anisotropies with crosslink modulation (Fig. 6) are consistent with decreased alignment of the collagen dipole moments within fibrils. Interestingly, based on these findings alone, it would be expected that even for the IOX2 modulated group, the SHG conversion efficiency (Fig. 4) would decrease due to decreased organization and increased phase-mismatch. However, these effects are likely overcome by the increased harmonophore density that occurs upon enhanced crosslinking by IOX2, as in the dense and completely aligned limiting case, the SHG conversion scales as the square of concentration. Thus, it is important to have this full suite of analysis tools to characterize collagen architecture changes upon crosslink modulation.

We can place our findings in the context of several human studies. First, we point out that these models of crosslink modulation are relevant to human IPF, where for example, Tschumperlin and co-workers found that LOXL1 and LOXL2 gene and protein levels were increased in IPF samples.^4^^,^^15^ Additionally, Jones showed that crosslink enhancement in the same spheroid models used here led to increased mechanical stiffness, which is consistent with the IPF clinical presentation.^4^ In our previous work on human IPF tissues, we found higher fiber alignment in IPF tissues (through machine learning-based image analysis), although normal fibers themselves were straighter.^6^ This is consistent with structural data in human IPF tissues as well.^7^ Interestingly, while the collagen in the spheroids was produced by IPF fibroblasts, those without crosslink modulation were the straightest. We also found that human IPF tissues had decreased underlying organization (sub-micron) through wavelength-dependent optical scattering measurements and reduced phase-matching based on SHG directional analysis.^15^ Further, the IPF tissues had reduced anisotropy and chirality compared to normal lung, similar to the spheroids with crosslink modulation used here. Collectively, these experiments demonstrate the need for a series of measurements probing all levels of collagen architecture to interrogate the role of crosslinking alterations in IPF in *in vitro* models and *ex vivo* tissues. This analysis can then provide insight into disease etiology and progression.

## 5 Conclusions

We have shown that SHG metrics based on coherence and polarization analysis are powerful for probing the collagen macro/supramolecular alterations in 3D *in vitro* models of IPF with enhanced and inhibited crosslinking. These models have relevance to human IPF as it has been established that LOX expression is up-regulated in human disease, resulting in increased crosslinking and corresponding matrix stiffness. Moreover, the use of multiple SHG metrics can provide structural information not possible by other methods. While there are no SHG metrics uniquely related to crosslinking, using a suite of SHG tools, we found changes in structure consistent with those expected by LOX modulation. Moreover, these findings using *in vitro* models produced under well-controlled conditions were analogous to those in our previous work on *ex vivo* IPF tissues. Collectively, these measurements using 3D models and SHG analysis provide great promise for understanding disease etiology of IPF as the approach permits extensive hypothesis testing of cellular and molecular factors relevant to this disease. Identifying these features in a known *in vitro* system is a critical step for eventual translation to *in vivo* imaging diagnostics.

## References

[1] Selman M. et al., “Idiopathic pulmonary fibrosis: prevailing and evolving hypotheses about its pathogenesis and implications for therapy,” Ann. Intern Med. 134, 136–151 (2001). 10.7326/0003-4819-134-2-200101160-00015 11177318

[r2] Butler M. W. Keane M. P., “The role of immunity and inflammation in IPF pathogenesis,” in Idiopathic Pulmonary Fibrosis: A Comprehensive Clinical Guide, Meyer K. C. Nathan S. D., Eds., pp. 97–131, Springer International Publishing, Cham (2019).

[3] Meyer K. C. Nathan S. D., Idiopathic Pulmonary Fibrosis: A Comprehensive Clinical Guide, Humana Press, New York (2014).

[4] Jones M. G. et al., “Nanoscale dysregulation of collagen structure-function disrupts mechano-homeostasis and mediates pulmonary fibrosis,” eLife 7, e36354 (2018). 10.7554/eLife.36354 29966587 PMC6029847

[5] Wittekind D., “Traditional staining for routine diagnostic pathology including the role of tannic acid. 1. Value and limitations of the hematoxylin-eosin stain,” Biotech. Histochem. 78, 261–270 (2003). BIHIEU 1052-0295 10.1080/10520290310001633725 14989644

[6] Tilbury K. et al., “Second harmonic generation microscopy analysis of extracellular matrix changes in human idiopathic pulmonary fibrosis,” J. Biomed. Opt. 19, 086014 (2014). JBOPFO 1083-3668 10.1117/1.JBO.19.8.086014 25134793 PMC4137064

[7] James D. S. et al., “Probing ECM remodeling in idiopathic pulmonary fibrosis via second harmonic generation microscopy analysis of macro/supramolecular collagen structure,” J. Biomed. Opt. 25, 014505 (2019). JBOPFO 1083-3668 10.1117/1.JBO.25.1.014505 PMC7008503 31785093

[r8] Campbell K. R. et al., “Polarization-resolved second harmonic generation imaging of human ovarian cancer,” J. Biomed. Opt. 23, 066501 (2018). JBOPFO 1083-3668 10.1117/1.JBO.23.6.066501 PMC5998835 29900704

[r9] Tilbury K. et al., “Differentiation of Col I and Col III isoforms in stromal models of ovarian cancer by analysis of second harmonic generation polarization and emission directionality,” Biophys. J. 106, 354–365 (2014). BIOJAU 0006-3495 10.1016/j.bpj.2013.10.044 24461010 PMC3907237

[10] Campbell K. R. Campagnola P. J., “Wavelength-dependent second harmonic generation circular dichroism for differentiation of Col I and Col III isoforms in stromal models of ovarian cancer based on intrinsic chirality differences,” J. Phys. Chem. B 121, 1749–1757 (2017). JPCBFK 1520-6106 10.1021/acs.jpcb.6b06822 28170263 PMC5494177

[11] Rodríguez C. et al., “Regulation of lysyl oxidase in vascular cells: lysyl oxidase as a new player in cardiovascular diseases,” Cardiovasc. Res. 79, 7–13 (2008). CVREAU 0008-6363 10.1093/cvr/cvn102 18469024

[r12] Barry-Hamilton V. et al., “Allosteric inhibition of lysyl oxidase-like-2 impedes the development of a pathologic microenvironment,” Nat. Med. 16, 1009–1017 (2010).1078-8956 10.1038/nm.2208 20818376

[r13] Kirk J. M. et al., “Biochemical evidence for an increased and progressive deposition of collagen in lungs of patients with pulmonary fibrosis,” Clin. Sci. (Lond.) 70, 39–45 (1986). 10.1042/cs0700039 3943276

[14] Fulmer J. D. et al., “Collagen concentration and rates of synthesis in idiopathic pulmonary fibrosis,” Am. Rev. Respir. Dis. 122, 289–301 (1980). ARDSBL 0003-0805 10.1164/arrd.1980.122.2.289 7416606

[15] Tjin G. et al., “Lysyl oxidases regulate fibrillar collagen remodelling in idiopathic pulmonary fibrosis,” Dis. Model. Mech. 10, 1301–1312 (2017). 10.1242/dmm.030114 29125826 PMC5719253

[16] Yao L. et al., “Paracrine signalling during ZEB1-mediated epithelial-mesenchymal transition augments local myofibroblast differentiation in lung fibrosis,” Cell Death Differ. 26, 943–957 (2019). 10.1038/s41418-018-0175-7 30050057 PMC6252080

[17] Chen X. et al., “Second harmonic generation microscopy for quantitative analysis of collagen fibrillar structure,” Nat. Protoc. 7, 654–669 (2012).1754-2189 10.1038/nprot.2012.009 22402635 PMC4337962

[18] Lien C. H. et al., “Precise, motion-free polarization control in Second Harmonic Generation microscopy using a liquid crystal modulator in the infinity space,” Biomed. Opt. Express 4, 1991–2002 (2013). BOEICL 2156-7085 10.1364/BOE.4.001991 24156059 PMC3799661

[19] LaComb R. et al., “Phase matching considerations in second harmonic generation from tissues: effects on emission directionality, conversion efficiency and observed morphology,” Opt. Commun. 281, 1823–1832 (2008). OPCOB8 0030-4018 10.1016/j.optcom.2007.10.040 19343083 PMC2390911

[20] Hall G. et al., “Experimental and simulation study of the wavelength dependent second harmonic generation of collagen in scattering tissues,” Opt. Lett. 39, 1897–1900 (2014). OPLEDP 0146-9592 10.1364/OL.39.001897 24686633 PMC4487653

[21] Nadiarnykh O. et al., “Alterations of the extracellular matrix in ovarian cancer studied by Second Harmonic Generation imaging microscopy,” BMC Cancer 10, 94 (2010). BCMACL 1471-2407 10.1186/1471-2407-10-94 20222963 PMC2841668

[22] Lacomb R. Nadiarnykh O. Campagnola P. J., “Quantitative SHG imaging of the diseased state Osteogenesis Imperfecta: experiment and Simulation,” Biophys. J. 94, 4504–4514 (2008). BIOJAU 0006-3495 10.1529/biophysj.107.114405 18281387 PMC2480682

[23] Nadiarnykh O. Campagnola P. J., “Retention of polarization signatures in SHG microscopy of scattering tissues through optical clearing,” Opt. Express 17, 5794–5806 (2009). OPEXFF 1094-4087 10.1364/OE.17.005794 19333348 PMC4487673

[24] Duboisset J. et al., “Generic model of the molecular orientational distribution probed by polarization-resolved second-harmonic generation,” Phys. Rev. A 85, 043829 (2012). 10.1103/PhysRevA.85.043829

[25] Plotnikov S. V. et al., “Characterization of the myosin-based source for second-harmonic generation from muscle sarcomeres,” Biophys. J. 90, 693–703 (2006). BIOJAU 0006-3495 10.1529/biophysj.105.071555 16258040 PMC1367074

[26] Gilkes D. M. et al., “Hypoxia-inducible factor 1 (HIF-1) promotes extracellular matrix remodeling under hypoxic conditions by inducing P4HA1, P4HA2, and PLOD2 expression in fibroblasts,” J. Biol. Chem. 288, 10819–10829 (2013). JBCHA3 0021-9258 10.1074/jbc.M112.442939 23423382 PMC3624462

[27] Schietke R. et al., “The lysyl oxidases LOX and LOXL2 are necessary and sufficient to repress E-cadherin in hypoxia: insights into cellular transformation processes mediated by HIF-1,” J. Biol. Chem. 285, 6658–6669 (2010). JBCHA3 0021-9258 10.1074/jbc.M109.042424 20026874 PMC2825461

[28] Bredfeldt J. S. et al., “Computational segmentation of collagen fibers from second-harmonic generation images of breast cancer,” J. Biomed. Opt. 19, 016007, doi 10.1117/1.JBO.19.1.016007 (2014). JBOPFO 1083-3668 10.1117/1.JBO.19.1.016007 PMC3886580 24407500

[29] Campbell K. R. Campagnola P. J., “Assessing local stromal alterations in human ovarian cancer subtypes via second harmonic generation microscopy and analysis,” J. Biomed. Opt. 22, 116008 (2017). JBOPFO 1083-3668 10.1117/1.JBO.22.11.116008 PMC5847644 29188658

[30] Brereton C. et al., “Pseudohypoxic HIF pathway activation dysregulates collagen structure-function in human lung fibrosis,” bioRxiv (2021). 10.7554/eLife.69348 PMC8860444 35188460

[31] Campagnola P., “Second harmonic generation imaging microscopy: applications to diseases diagnostics,” Anal. Chem. 83, 3224–3231 (2011). ANCHAM 0003-2700 10.1021/ac1032325 21446646 PMC3104727

[32] Raghu G. et al., “Extracellular matrix in normal and fibrotic human lungs,” Am. Rev. Respir. Dis. 131, 281–289 (1985). ARDSBL 0003-0805 10.1164/arrd.1985.131.2.281 3882034

[33] Kong J. Yu S., “Fourier transform infrared spectroscopic analysis of protein secondary structures,” Acta Biochim. Biophys. Sin. (Shanghai) 39, 549–559 (2007). 10.1111/j.1745-7270.2007.00320.x 17687489

[34] Paschalis E. P. et al., “Spectroscopic characterization of collagen cross-links in bone,” J. Bone Miner. Res. 16, 1821–1828 (2001). JBMREJ 0884-0431 10.1359/jbmr.2001.16.10.1821 11585346

[35] Tilbury K. B. et al., “Stromal alterations in ovarian cancers via wavelength dependent Second Harmonic Generation microscopy and optical scattering,” BMC Cancer 17(1), 102 (2017). 10.1186/s12885-017-3090-2 28166758 PMC5294710

[36] Marturano J. E. et al., “Characterization of mechanical and biochemical properties of developing embryonic tendon,” Proc. Natl. Acad. Sci. U. S. A. 110, 6370–6375 (2013). 10.1073/pnas.1300135110 23576745 PMC3631620

[r37] Marturano J. E. et al., “Lysyl oxidase-mediated collagen crosslinks may be assessed as markers of functional properties of tendon tissue formation,” Acta Biomater. 10, 1370–1379 (2014). 10.1016/j.actbio.2013.11.024 24316363 PMC4053294

[r38] Lee P. F. et al., “Angiogenic responses are enhanced in mechanically and microscopically characterized, microbial transglutaminase crosslinked collagen matrices with increased stiffness,” Acta Biomater. 9, 7178–7190 (2013). 10.1016/j.actbio.2013.04.001 23571003 PMC3749884

[r39] Tan H. Y. et al., “Characterizing the morphologic changes in collagen crosslinked-treated corneas by Fourier transform-second harmonic generation imaging,” J. Cataract Refract. Surg. 39, 779–788 (2013). 10.1016/j.jcrs.2012.11.036 23608570

[r40] Bueno J. M. Ávila F. J. Martínez-García M. C., “Quantitative analysis of the corneal collagen distribution after *in vivo* cross-linking with second harmonic microscopy,” Biomed. Res. Int. 2019, 3860498 (2019). 10.1155/2019/3860498 30756083 PMC6348900

[41] Lutz V. et al., “Impact of collagen crosslinking on the second harmonic generation signal and the fluorescence lifetime of collagen autofluorescence,” Skin Res. Technol. 18, 168–179 (2012). 10.1111/j.1600-0846.2011.00549.x 21564311

[42] Tuer A. E. et al., “Hierarchical model of fibrillar collagen organization for interpreting the second-order susceptibility tensors in biological tissue,” Biophys. J. 103, 2093–2105 (2012). BIOJAU 0006-3495 10.1016/j.bpj.2012.10.019 23200043 PMC3512050

[43] Golaraei A. et al., “Complex susceptibilities and chiroptical effects of collagen measured with polarimetric second-harmonic generation microscopy,” Sci. Rep. 9, 12488 (2019). SRCEC3 2045-2322 10.1038/s41598-019-48636-w 31462663 PMC6713739

[44] Schmeltz M. et al., “Circular dichroism second-harmonic generation microscopy probes the polarity distribution of collagen fibrils,” Optica 7, 1469–1476 (2020). 10.1364/OPTICA.399246

